# Evolution of Clinical Fragility and Medication Burden in Children with Medical Complexity: A Longitudinal Cohort Study

**DOI:** 10.3390/children13081012

**Published:** 2026-07-30

**Authors:** Anna Zanin, Fernando Baratiri, Gloria Brigiari, Barbara Roverato, Daniele Mengato, Laura Camuffo, Dario Gregori, Francesca Venturini, Franca Benini

**Affiliations:** 1 Centre de Coordination Interdisciplinaire et de Soins des Maladies Rares et Complexes de l’Enfant et de l’Adolescent, Département de la Femme, de l’Enfant et de l’Adolescent, Service de Pédiatrie Générale, Hôpital des Enfants, 1211 Geneva, Switzerland; 2 Palliative Care and Pain Service, Department of Women’s and Children’s Health, University of Padua, 35128 Padua, Italy; 3 Department of Pediatrics, Azienda Ulss3 Serenissima, Ospedale Dell’Angelo, Mestre-Venezia, 30174 Venice, Italy; 4 Unit of Biostatistics, Epidemiology and Public Health, Department of Cardiac, Vascular Sciences and Public Health, University of Padova, 35128 Padua, Italy; 5 BIOSTAT-X Biostatistics & AI for Biomedical Discovery, Pediatric Research Institute (IRP) “Città della Speranza”, 35127 Padova, Italy; 6 PhD Program in Translation Specialistic Medicine “G.B. Morgagni”, Curriculum “Biostatistics and Clinical Epidemiology”, University of Padua, 35128 Padua, Italy; 7 Hospital Pharmacy Department, Padua University Hospital, 35128 Padua, Italy

**Keywords:** children with medical complexity (CMC), pediatric palliative care, medication burden, polypharmacy, healthcare needs

## Abstract

**Highlights:**

**What are the main findings?**

- Children with medical complexity showed increasing care and treatment complexity over time, with higher ACCAPED scores, more medications, and more daily administrations.
- The number of prescribed drugs strongly correlated with overall healthcare needs.

**What are the implications of the main findings?**

- Medication regimen characteristics were strongly associated with a child’s underlying care complexity.
- Tracking medication burden may contribute to understanding the evolution of care complexity in children.

**Abstract:**

Background/Objectives: Children with medical complexity (CMC) often suffer from chronic or evolving conditions that require increasingly complex treatment regimens over time, resulting in increased therapeutic and care burdens, costs, and the risk of adverse effects and medication errors. The study aimed to describe the variation in healthcare needs, medication regimens and medication burden over time and to describe the relationship between the number of drugs and healthcare needs. Methods: In this cohort study, conducted at the Pediatric Palliative Care Center of Padua, Italy, two repeated assessments were performed, respectively, in October 2021 (t_0_) and in February 2023 (t_1_). A total of 169 patients aged ≤23 years receiving at least one prescribed medication were enrolled. Longitudinal analyses were performed in the 147 patients with complete assessments at both time points. Data were collected from medical records and caregiver interviews. Drug costs were collected from the Italian Medicines Agency. Changes between baseline and follow-up were assessed using paired statistical tests (Wilcoxon signed-rank test for continuous variables and McNemar test for categorical variables). The associations between the number of drugs and the ACCAPED scale (Accertamento dei Bisogni Clinico Assistenziali Complessi in Cure Palliative Pediatriche) were assessed using linear regression models. Results: The study analyzed treatment regimens of 147 patients with a median age of 12.1 years at t_0_ and 14.1 at t_1_. The prevalence of patients with medication burden (42% vs. 50%, *p* = 0.022), do-not-resuscitate order (20% vs. 27%, *p* = 0.027) and an ACCAPED score > 50 (57% vs. 66%, *p* = 0.012) significantly increased on the second assessment. Also, the number of total daily administrations (5 [3–12] vs. 6 [3–14], *p* = 0.019) and the number of medications (4 [2–7] vs. 5 [3–8], *p* = 0.013) significantly increased. However, polypharmacy and the median daily cost per patient did not significantly increase between t_0_ and t_1_. The association between the number of drugs and the ACCAPED score showed that each additional prescribed drug is associated with an average increase of 3.2 points in the ACCAPED score (95% CI: 2.6, 3.9; *p* < 0.001). Conclusions: Over time, the degree of complexity in our population increased significantly, as evidenced by the increased healthcare needs, treatment regimens and burden on families. The number of prescribed drugs showed a strong and statistically significant relationship with patient care complexity. Treatment regimen characteristics were strongly associated with patient care complexity. Future studies should evaluate whether these pharmacological variables may have a role in supporting clinical assessment.

## 1. Introduction

The management of Children with Medical Complexity (CMC) represents one of the most significant challenges in modern pediatrics, creating a high-stakes environment where clinical fragility and caregiver burden are inextricably linked [1,2]. Characterized by multi-organ chronic conditions and functional limitations, these children require high healthcare resource utilization and multi-dimensional care, often coordinated by Pediatric Palliative Care (PPC) teams or specialized complex care centers [3].

Achieving symptom control represents a key objective of disease management and frequently necessitates the implementation of complex medication regimens (CMRs) [4,5,6].

For parents and caregivers, the transition from hospital to home care entails a substantial shift in responsibilities, requiring them to assume tasks typically performed by healthcare professionals, including medication preparation and administration, without the structured support available in clinical settings [7]. This largely unsupported role places considerable demands on caregivers, who must independently manage complex medication schedules and administration procedures. In the United States, between 2002 and 2012, an out-of-hospital medication error involving a child younger than six years occurred approximately every eight minutes, underscoring the magnitude of this challenge [8]. The involvement of multiple caregivers, including school personnel and respite care providers, further increases the complexity of medication management. Consequently, the risk of administration discrepancies—such as errors in dose, timing, or route of administration—is substantial. This burden is amplified when high-risk medications are prescribed or when medications must be administered via non-standard routes, such as enteral feeding tubes, which require ongoing technical management and may result in serious clinical consequences if doses are omitted or administered incorrectly [9,10,11].

Ultimately, the need to achieve adequate symptom control often results in chronic polypharmacy, which is associated with an increased risk of adverse drug events, including drug–drug interactions and medication administration errors [12,13]. Several tools have been developed to quantify pharmacotherapeutic complexity, including the Medication Regimen Complexity Index (MRCI), which has been applied in pediatric populations [14,15,16,17,18]. Although comprehensive, these instruments require detailed manual assessment of dosage forms, administration frequencies, and additional dosing instructions. Consequently, their routine use in clinical practice may be limited, particularly in primary care settings. This has driven interest in identifying simpler pharmacological indicators that correlate with increasing care complexity.

The aim of this longitudinal study was to evaluate the evolution of clinical complexity and medication burden in a cohort of CMC over a 16-month period, identifying pharmacological characteristics associated with clinical complexity over time.

## 2. Materials and Methods

### 2.1. Study Design and Population

This longitudinal cohort study was conducted at the Pediatric Palliative Care Center of Padua, Italy, between October 2021 (time of the first survey, t_0_) and February 2023 (time of the second survey, t_1_). All patients up to 23 years of age with at least one prescribed medication were eligible. Of the 169 patients initially assessed at baseline, 147 had complete data available at both time points and were included in the longitudinal analyses. Of the 22 patients excluded, 15 had died at the time of t_1_, 5 had been discharged from the PPC service, and 2 did not meet the inclusion criteria.

Demographics and pharmacological data, including information about patients’ baseline conditions, principal symptoms and pharmacological therapies, were collected from the electronic medical charts.

### 2.2. Clinical and Pharmacological Assessment

- **Clinical Complexity:** Clinical needs and care complexity were evaluated using the ACCAPED scale (Accertamento dei Bisogni Clinico Assistenziali Complessi in Cure Palliative Pediatriche), a validated Italian multidimensional tool specifically designed for pediatric palliative care [18]. The scale evaluates five clinical and care domains: clinical status and fragility, nursing care needs, psychological and social needs, technological dependency, and ethical/end-of-life decisions. The cumulative score ranges from 0 to 100, with scores ≥ 50 indicating high complexity needs and eligibility for specialized PPC services. In this study, ACCAPED scores were assessed and recorded by the clinical team (attending pediatricians and specialized PPC nurses) during routine clinical evaluations and structured caregiver interviews at the two timepoints.
- **Polypharmacy:** Polypharmacy was defined as the concurrent daily administration of five or more ≥ 5 scheduled prescription medications, while excessive polypharmacy was defined as the concurrent administration of ten or more ≥ 10 scheduled medications. These cut-offs were chosen based on the established literature and previous studies in pediatric populations and children with medical complexity. Only routinely scheduled maintenance therapies were included in these definitions, whereas as-needed (PRN) medications, dietary supplements, and medical devices were evaluated separately to avoid overestimating baseline daily therapeutic burden [19,20].
- **Medication Burden:** This composite definition was chosen to capture both pharmacological intensity and the practical burden of treatment management. While polypharmacy reflects the number of prescribed medications, repeated scheduled nighttime administrations represent an additional organizational burden for caregivers. The threshold of at least two nighttime administrations (20:00 to 07:59) was selected to identify patients requiring recurrent overnight medication management rather than an isolated bedtime dose, based on our previous experience in pediatric palliative care [4,19].
- **MRCI:** Regimen complexity was quantified using the Medication Regimen Complexity Index (MRCI), a validated tool for patients experiencing polypharmacy [16]. The MRCI measures overall complexity beyond simple drug counts across three distinct sections: Section A (dosage forms), Section B (dosing frequency), and Section C (additional administration instructions, such as relationship to meals or special preparation requirements). Higher total scores reflect greater pharmacotherapeutic complexity. In this study, MRCI scores were calculated by hospital pharmacists who extracted and reviewed medication data directly from electronic medical charts and cross-verified them with caregiver-reported schedules and with pharmacy reports.

### 2.3. Statistical Analysis

Descriptive statistics were reported as the median with first and third quartiles (Q1–Q3) for continuous variables and absolute numbers and percentages for categorical data. Comparisons were performed with the Wilcoxon signed rank test with continuity correction for continuous variables and McNemar’s Chi-squared test with continuity correction for categorical variables. For each comparison, we also report an effect size with its 95% confidence interval: the median paired difference between the two assessments (Hodges–Lehmann estimate) for continuous variables, and the absolute change in prevalence, in percentage points, for categorical variables. Associations between pharmacological indicators (number of prescribed drugs and MRCI) and ACCAPED score were assessed using linear regression models with Huber–White robust standard errors to account for repeated observations within patients. Multivariable models were adjusted for age and diagnostic category. Because the aim was to describe associations rather than to develop or validate a prediction model, model-performance metrics such as explained variance or calibration measures were not considered primary outputs. To assess the linearity assumption between the number of prescribed medications and ACCAPED score, restricted cubic spline models with three knots were fitted using the rms package. Non-linearity was formally tested using Wald statistics for the nonlinear spline components. In the absence of significant non-linearity, a linear term was retained in the final model. The results of these analyses were reported as beta coefficients and 95% CI. Statistical significance was defined as *p* < 0.05. Missing data were not imputed. Longitudinal analyses were restricted to patients with complete assessments at both time points, and all comparisons and regression models were fitted on complete cases, using all patients with non-missing values for the variables involved. The number of observations contributing to each analysis is reported in the corresponding table, and missing values are shown as a separate “Unknown” category in the descriptive tables.

Statistical analyses were performed using the R System version 4.3.3 (version 4.3.3; R Foundation for Statistical Computing, Vienna, Austria) [21], package rms.

## 3. Results

### 3.1. Population and Clinical Status

The study tracked 147 patients over a 16-month interval (for a complete description of the cohort at t_0_, see Annexes), with a median age advancing from 12.1 [6.1–14.9] years at t_0_ to 14.1 [8.0–16.9] years at t_1_.

Almost all patients had a congenital or perinatal condition (92.5%). The main diagnostic category was “neurological disorders”, which accounts for almost half of the population (42.2%), followed by “muscular–skeletal disorders” (29.3%) and “genetic–metabolic disorders” (16.3%). The remaining categories “respiratory disorders”, “oncological disorders” and “cardiovascular disorders” represent 7.5%, 3.4% and 1.4% of the population. Notably, when we analyzed the main diagnostic category of the 15 patients excluded from the comparison because they had died at the time of t_1_, we found that 6 of them (40%) were diagnosed with an “oncological disorder”.

During this period, the cohort exhibited a marked shift toward higher clinical fragility (Table 1). The prevalence of patients categorized with high-complexity needs—defined by an ACCAPED score ≥ 50—rose significantly from 57% to 66% (*p* = 0.012). The trend was reflected by the significant rise in the median ACCAPED score from 54 (37, 69) to 61 (45, 72) (*p* < 0.0001). This clinical decline was mirrored by a significant increase in the establishment of “Do Not Resuscitate” (DNR) orders, which grew from 20% to 27% (*p* = 0.027).

We also observed an increase in oxygen dependency as an indicator of clinical deterioration (32% vs. 41%, *p* = 0.008).

### 3.2. Shifts in Therapeutic Intensity and Reorientation of Care

The therapeutic demand placed on families and caregivers intensified alongside the clinical decline, as shown in Table 2. The medication burden, a composite measure of polypharmacy and frequent nighttime administrations, escalated from 42% at the first survey to 50% at the second (*p* = 0.022). This was supported by a statistically significant rise in the volume of care:

- The median number of total daily administrations increased from 5 [3–12] to 6 [3–14] (*p* = 0.019).
- The median number of medications per patient rose from 4 [2–7] to 5 [3–8] (*p* = 0.013).

**Figure 1:**
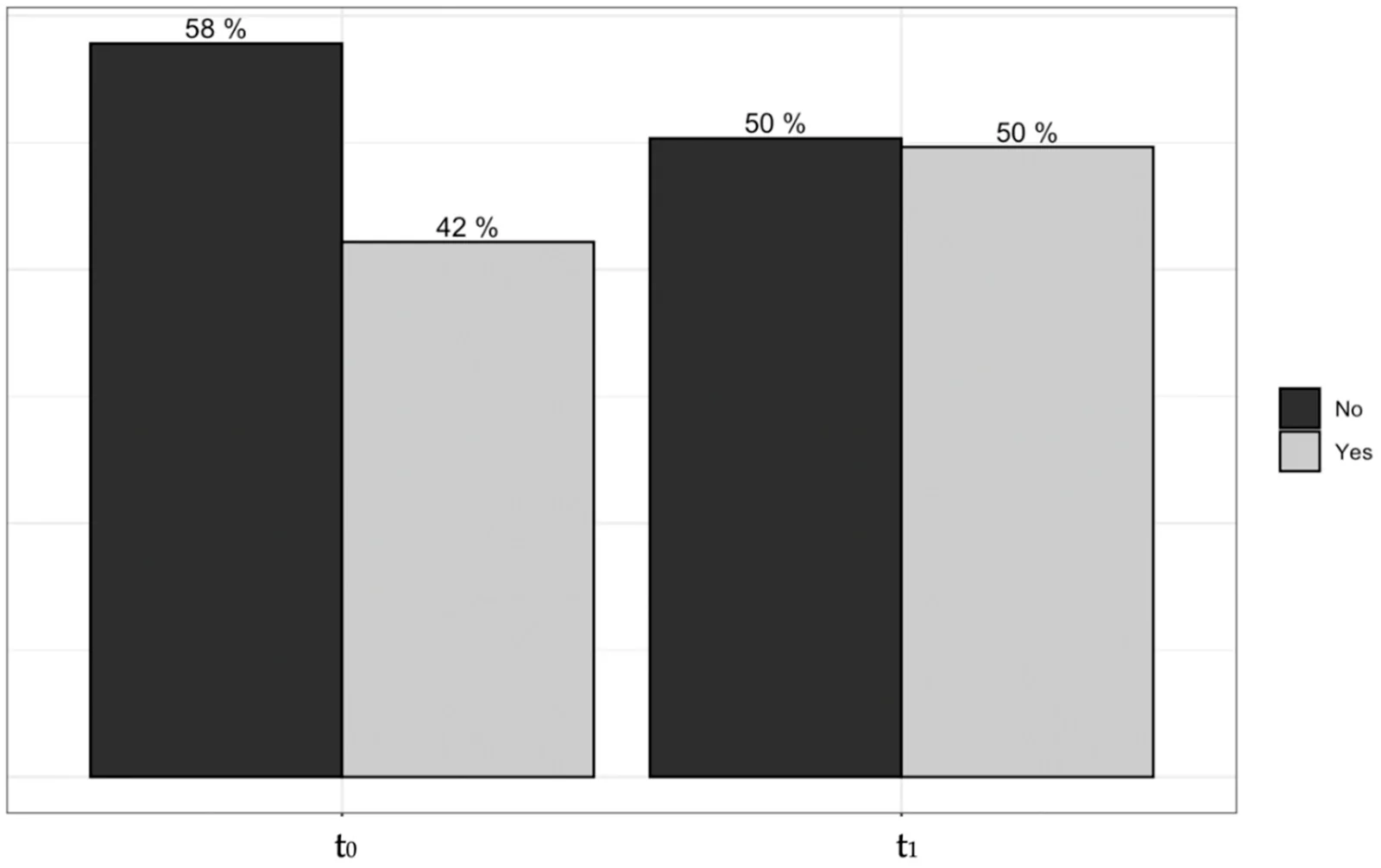
The prevalence of medication burden at the two timepoints.

Notably, while the raw number of drugs and administrations grew, the broad categorical prevalence of polypharmacy and the median daily financial cost per patient remained relatively stable between the two time points, suggesting that the “burden” grew more through the complexity of administration schedules than through the introduction of high-cost new agents (Figure 1).

### 3.3. Association Between Medication Count and Clinical Complexity

**Figure 2:**
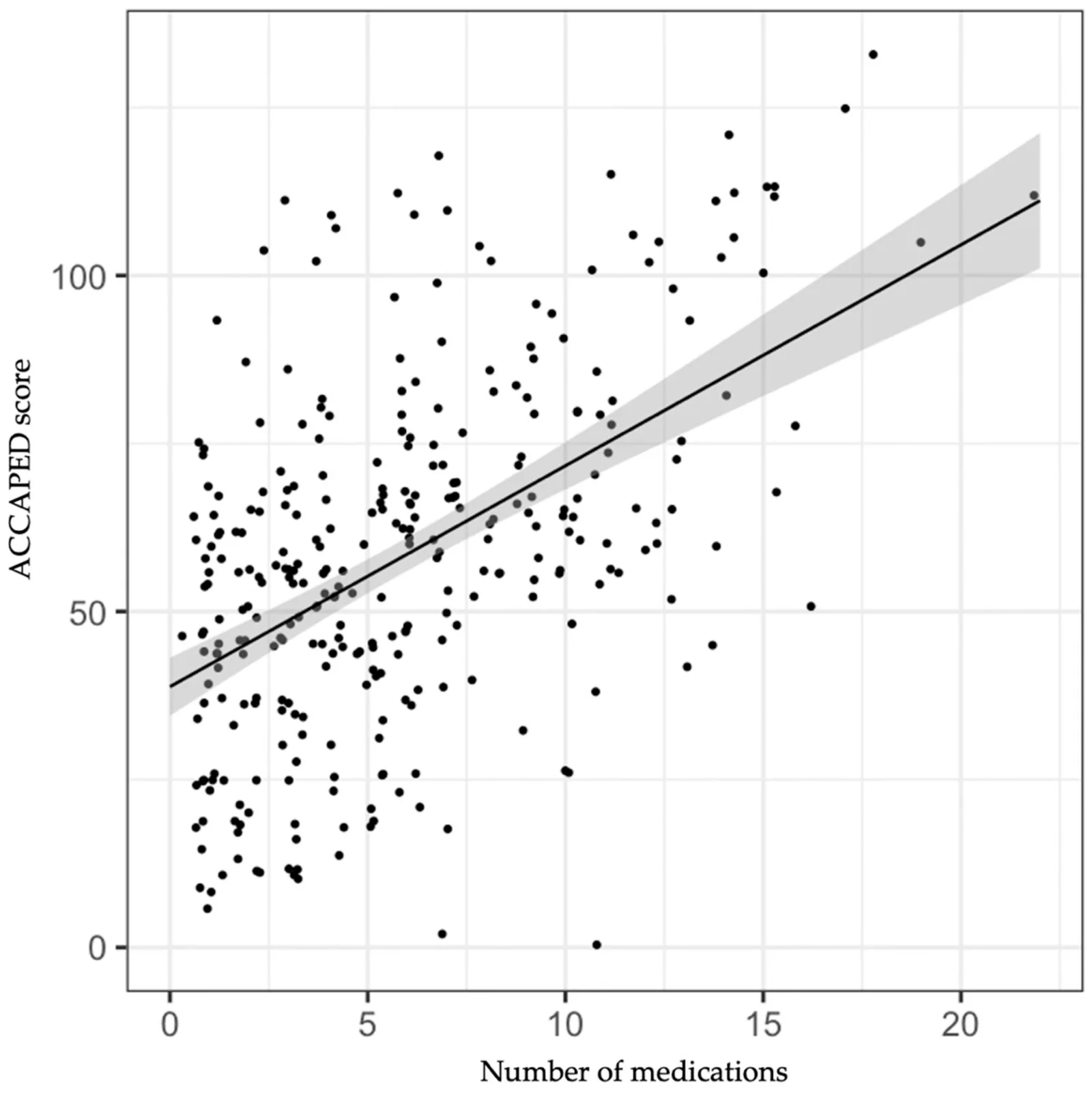
Association between number of prescribed medications and ACCAPED score. Scatterplot with fitted linear regression line and 95% confidence band (in grey).

**Figure 3:**
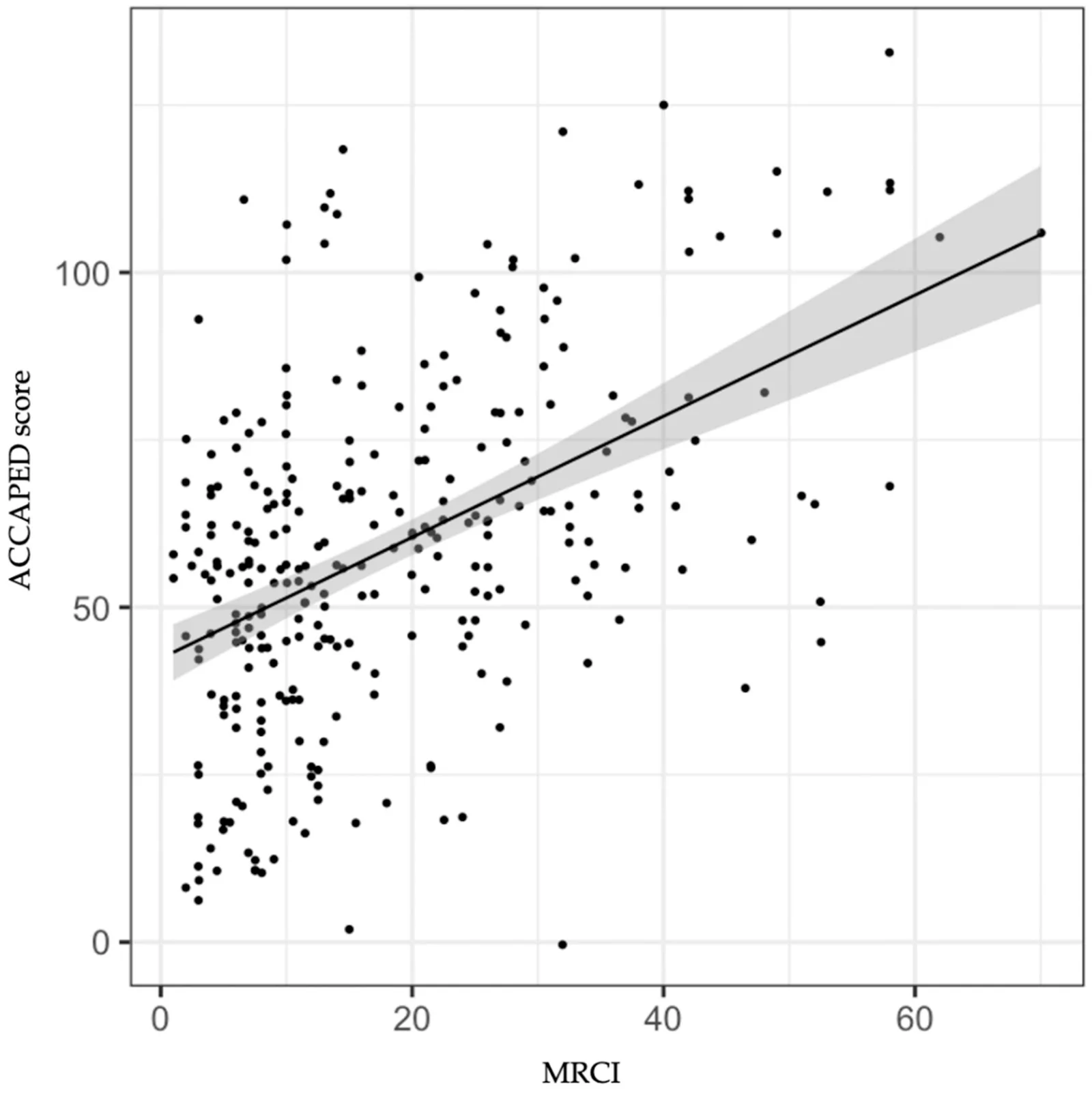
Association between Medication Regimen Complexity Index (MRCI) and ACCAPED score. Scatterplot with fitted linear regression line and 95% confidence band (in grey).

A primary finding of this study was the robust linear relationship between medication count and clinical complexity (Figure 2). In the unadjusted model, each additional drug was associated with a 3.2-point higher ACCAPED score (95% CI: 2.4 to 4.1; *p* < 0.001). After adjustment for age and diagnostic category, the association remained similar (adjusted beta = 3.1; 95% CI: 2.2 to 4.0; *p* < 0.001). The linearity assumption was formally assessed using restricted cubic splines. No evidence of non-linearity was detected (*p* for nonlinear component = 0.44), supporting the use of a linear model (Annexes). MRCI was also positively associated with ACCAPED score (Figure 3). The unadjusted beta coefficient was 0.88 (95% CI: 0.61 to 1.1; *p* < 0.001), and the association remained statistically significant after adjustment for age and diagnostic category (adjusted beta = 0.85; 95% CI: 0.54 to 1.2; *p* < 0.001).

## 4. Discussion

The present study demonstrates a progressive escalation in both clinical fragility and therapeutic intensity among children with medical complexity over a 16-month period. ACCAPED scores and indicators of clinical decline, including oxygen dependence and DNR orders, increased significantly, and this trajectory was accompanied by a measurable rise in medication burden and daily administration requirements. The observed associations between pharmacological indicators and ACCAPED score suggest that medication regimen information was strongly associated with care complexity. However, these results should be interpreted within an association framework and do not establish causality or predictive performance.

The multivariable analyses partially address the concern that the association between medication count and ACCAPED score could be explained by patient characteristics. After adjustment for age and diagnostic category, the association between medication count and ACCAPED score remained similar to the unadjusted estimate. MRCI showed the same pattern, with a statistically significant adjusted association. This supports the robustness of the association, but residual confounding by disease severity, functional status, and other unmeasured clinical features remains possible. Therefore, medication count appears to be consistently associated with higher clinical complexity. Whether this association may translate into clinically useful assessment strategies should be evaluated in prospective validation studies.

MRCI adds complementary information by capturing the practical complexity of medication regimens, including dosage forms, administration frequency, and instructions. In contrast, the raw number of prescribed medications is easier to retrieve from clinical records and may be useful as an initial flag in routine care. The increase in night-time administrations also highlights that medication burden is not limited to the number of drugs but includes the temporal organization of care and its impact on family workload. These findings support periodic, multidisciplinary medication reviews in CMC, especially when pharmacological regimens expand or become more difficult to administer.

To address these fragmented care patterns, innovative strategies are shifting toward a more collaborative model. This includes the integration of pharmacists as “quarterbacks” for the medical team [22], a move that has been shown to reduce the average number of medications and prevent costly adverse events [23,24]. Simultaneously, digital interventions like the Meds@HOME app are being co-designed with families to standardize care across the entire network of caregivers [25]. By synchronizing communication between parents and secondary caregivers, these tools aim to create a digital safety net, ensuring that complex medical routines are maintained with accuracy even as the child moves through different environments. Ultimately, improving the care of CMC requires recognizing that polypharmacy is not just a medical necessity, but a primary contributor to both health outcomes and the overall quality of life for the family [9].

The observed association between medication count and ACCAPED score suggests that simple pharmacological variables may reflect overall care complexity. However, because this was an observational study, our findings should not be interpreted as validating medication count as a screening or prediction tool. Future prospective studies should determine whether medication-related variables may complement existing multidimensional assessments in clinical practice.

This study has limitations that should be considered when interpreting the findings. First, it was conducted in a single specialized Pediatric Palliative Care center with a relatively limited sample size, which may limit generalizability to other healthcare settings, including primary care and non-specialized pediatric services. Second, the exclusion of 22 patients at follow-up introduces survivorship bias. Fifteen of these patients died and likely represented the most clinically fragile stratum of the initial cohort; therefore, the longitudinal increase in clinical complexity and medication burden observed among survivors may underestimate the true progression in the full baseline population. Third, part of the medication schedule data relied on caregiver-reported information, which may be subject to recall inaccuracies, although data were collected within routine clinical evaluations and cross-checked with medical records whenever possible. Finally, the analyses were designed to quantify associations, not to validate medication count or MRCI as prediction tools; external validation would be required before these measures could be used for risk stratification or decision-making.

## 5. Conclusions

Polypharmacy and medication burden are high in CMC and increase as the disease evolves. Our longitudinal analysis reveals a significant escalation in clinical complexity, manifested through intensified healthcare requirements, expanded therapeutic regimens, and an increasing burden on caregivers. We identified a robust, statistically significant correlation between the quantity of prescribed medications and overall care complexity. In this longitudinal cohort, medication count was strongly associated with clinical complexity. These findings support further investigation into whether simple pharmacological variables may complement multidimensional assessment tools after prospective validation.

## Figures and Tables

**Table 1:** Clinical characteristics of the study population at baseline and follow-up.

Descriptive Characteristics				
	t_0_ N = 147 ^1^	t_1_ N = 147 ^1^	Effect Size [CI 95%]	*p* Value ^2^
Do not resuscitate (DNR)			+6.1 pp (1.4 to 10.8)	0.027
Yes	30 (20%)	39 (27%)		
No	117 (80%)	108 (73%)		
ACCAPED				
Mean, (SD)	55, (26)	59, (25)		
Median (Q1, Q3)	54 (37, 69)	61 (45, 72)	3.50 (2.00 to 5.50)	<0.001
ACCAPED < 50	63 (43%)	50 (34%)		
ACCAPED ≥ 50	84 (57%)	97 (66%)	+8.8 pp (2.6 to 15.1)	0.012
Age (categories)				
Infant and preschool (0–5 yo)	36 (24%)	28 (19%)		
School (6–11 yo)	35 (24%)	37 (25%)		
Adolescent (12–17 yo)	59 (40%)	59 (40%)		
Adult	17 (12%)	23 (16%)		
Age				
Mean, (SD)	10.8, (5.6)	12.8, (5.6)		
Median (Q1, Q3)	12.1 (6.0, 14.9)	14.1 (8.0, 16.9)		
Age of disease				
Mean, (SD)	10.5, (5.7)	11.5, (5.7)		
Median (Q1, Q3)	12.0 (6.0, 15.0)	13.0 (7.0, 16.0)		
Oxygen therapy			+9.5 pp (3.2 to 15.9)	0.008
Yes	47 (32%)	61 (41%)		
No	100 (68%)	86 (59%)		
MRCI				
Mean, (SD)	18, (13)	18, (14)		
Median (Q1, Q3)	13 (8, 24)	14 (7, 27)	0.25 (0.00 to 1.00)	
Unknown	15	8		

^1^ Mean (SD) and Median (Q1, Q3); n (%). ^2^ Wilcoxon signed rank test with continuity correction; McNemar’s Chi-squared test with continuity correction.

**Table 2:** Medication regimen characteristics at baseline and follow-up.

Descriptive Characteristics				
	t0 N = 147 ^1^	t1 N = 147 ^1^	Effect Size [CI 95%]	*p* Value ^2^
Drug Administration			−1.4 pp (−3.2 to 0.5)	
Auto-administration	30 (20%)	28 (19%)		
Administration by the caregiver	117 (80%)	119 (81%)		
N Drugs				
Mean, (SD)	5.5, (4.0)	5.9, (4.1)		
Median (Q1, Q3)	4.0 (2.0, 7.0)	5.0 (3.0, 8.0)	0.50 (0.00 to 0.50)	0.013
Polypharmacotherapy (≥5/die)			+5.4 pp (−0.7 to 11.6)	
Yes	74 (50%)	82 (56%)		
No	73 (50%)	65 (44%)		
Maintenance therapy			0.50 (0.00 to 0.50)	
Mean, (SD)	5, (4)	5, (4)		
Median (Q1, Q3)	4 (2, 7)	5 (2, 7)		
As needed therapy			0.00 (0.00 to 0.00)	
Mean, (SD)	1, (1)	1, (1)		
Median (Q1, Q3)	0 (0, 1)	0 (0, 1)		
Unknown	1	0		
ATC Classes			0.00 (0.00 to 0.50)	
Mean, (SD)	3, (1)	3, (1)		
Median (Q1, Q3)	3 (2, 3)	3 (2, 3)		
Enteral/oral drugs			0.50 (0.00 to 0.50)	
Mean, (SD)	5, (4)	5, (4)		
Median (Q1, Q3)	4 (2, 7)	5 (2, 8)		
Intravenous/subcutaneous			0.00 (0.00 to 0.00)	
Mean, (SD)	0, (0)	0, (0)		
Median (Q1, Q3)	0 (0, 0)	0 (0, 0)		
Sublingual/rectal/transcutaneous			0.00 (0.00 to 0.00)	
Mean, (SD)	0, (1)	0, (1)		
Median (Q1, Q3)	0 (0, 0)	0 (0, 1)		
Daily administration (8–19 h)				
Mean, (SD)	5, (5)	6, (4)		
Median (Q1, Q3)	4 (2, 8)	4 (2, 8)	0.00 (0.00 to 0.50)	0.13
Night-time administration (20–7 h)				
Mean, (SD)	3, (3)	3, (3)		
Median (Q1, Q3)	2 (0, 3)	2 (0, 5)	0.00 (0.00 to 0.50)	0.013
Total daily administrations				
Mean, (SD)	8, (7)	9, (7)		
Median (Q1, Q3)	5 (3, 12)	6 (3, 14)	0.50 (0.00 to 1.00)	0.019
Medication burden				
Yes	62 (42%)	73 (50%)	+7.5 pp (1.8 to 13.2)	0.022
No	85 (58%)	74 (50%)		

^1^ Mean (SD) and Median (Q1, Q3); n (%). ^2^ Wilcoxon signed rank test with continuity correction; McNemar’s Chi-squared test with continuity correction.

## Data Availability

The data presented in this study are available on request from the corresponding author. The data are not publicly available due to privacy and ethical reasons.

## Abbreviations

The following abbreviations are used in this manuscript:

ACCAPED	Accertamento dei Bisogni Clinico Assistenziali Complessi in Cure Palliative Pediatriche
CMC	Children with Medical Complexities
CMR	Complex Medication Regimens
DNR	Do Not Resuscitate
PPC	Pediatric Palliative Care
